# The Fake IQ Test: a novel measure of self-reflection in major depressive disorder

**DOI:** 10.1192/bjo.2023.79

**Published:** 2023-06-08

**Authors:** Lindsey Marwood, Jess Kerr-Gaffney, Toby Wise, Rebecca Strawbridge, Adam M. Perkins, Anthony J. Cleare

**Affiliations:** Centre for Affective Disorders, Department of Psychological Medicine, Institute of Psychiatry, Psychology and Neuroscience, King's College London, UK; Department of Neuroimaging, Institute of Psychiatry, Psychology and Neuroscience, King's College London, UK; Centre for Affective Disorders, Department of Psychological Medicine, Institute of Psychiatry, Psychology and Neuroscience, King's College London, UK; and National Affective Disorders Service, South London and Maudsley NHS Foundation Trust, London, UK

**Keywords:** Depression, self-reflection, rumination, functional magnetic resonance imaging, imaging

## Abstract

**Background:**

Excessive negative self-referential processing plays an important role in the development and maintenance of major depressive disorder (MDD). Current measures of self-reflection are limited to self-report questionnaires and invoking imagined states, which may not be suitable for all populations.

**Aims:**

The current study aimed to pilot a new measure of self-reflection, the Fake IQ Test (FIQT).

**Method:**

Participants with MDD and unaffected controls completed a behavioural (experiment 1, *n* = 50) and functional magnetic resonance imaging version (experiment 2, *n* = 35) of the FIQT.

**Results:**

Behaviourally, those with MDD showed elevated negative self-comparison with others, higher self-dissatisfaction and lower perceived success on the task, compared with controls; however, FIQT scores were not related to existing self-report measures of self-reflection. In the functional magnetic resonance imaging version, greater activation in self-reflection versus control conditions was found bilaterally in the inferior frontal cortex, insula, dorsolateral prefrontal cortex, motor cortex and dorsal anterior cingulate cortex. No differences in neural activation were found between participants with MDD and controls, nor were there any associations between neural activity, FIQT scores or self-report measures of self-reflection.

**Conclusions:**

Our results suggest the FIQT is sensitive to affective psychopathology, but a lack of association with other measures of self-reflection may indicate that the task is measuring a different construct. Alternatively, the FIQT may measure aspects of self-reflection inaccessible to current questionnaires. Future work should explore relationships with alternative measures of self-reflection likely to be involved in perception of task performance, such as perfectionism.

## Self-reflection and depression

Self-reflection refers to the ability to think about one's own thoughts, feelings and actions. It can be seen as a dimensional construct, encompassing both positive forms (e.g. self-reassurance and compassion) and negative or maladaptive forms (e.g. excessive rumination and worry).^1^ Maladaptive forms of self-reflection play a major role in the development and course of mood and anxiety disorders.^2^ Prospective studies have demonstrated that rumination predicts the duration and severity of depressive symptoms, as well as the onset of new depressive episodes.^3,4^ Further, reductions in self-criticism during cognitive therapy predict treatment response in those with major depressive disorder (MDD).^5^ Development of objective measures of self-reflection may help to provide a better understanding of the development and maintenance of disordered thinking in MDD.

Self-reflective cognitions are conventionally assessed by self-report questionnaires. However, these rely on semantic autobiographical memory, and may be particularly inaccurate and insensitive to change in clinical populations with decreased insight.^6^ Other studies have used neuroimaging paradigms, whereby participants engage in imaginative states of self-referential thought; for example, by imagining scenarios of personal failures and mistakes or hopes and aspirations.^7,8^ However, these paradigms rely on the assumption that internally imagined states are equivalent to those produced by external stimuli. Although there is some evidence of overlap between real and imagined states, the validity of this design has been brought into question.^9^ Further, there are likely to be individual differences in one's ability to realistically imagine certain states, especially in certain patient groups with limited insight or alexithymia.^10,11^

A potential solution to these limitations is to use a more objective task, where participants engage in a genuine situation involving self-reflection. Nuttin and Greenwald^12^ created such a test, whereby participants were asked to judge pairs of shapes on various visual properties (e.g. which shape is bigger). The task was portrayed as an intelligence task, but was really an implicit measure of self-reflection; the task was in fact impossible, and shapes were equal on the queried visual property. In the original experiment, participants were split into ‘pessimists’ and ‘optimists’, and in a second experiment, ‘depressives’ and ‘manics’; in both cases, the former group was more likely to overestimate failures.^12^ The findings demonstrate that individuals perceive successes and failures in the context of a pre-established personality trait-like conception of the self, independent of actual task performance.

## The Fake IQ Test

The Fake IQ Test (FIQT) was created as an adapted version of Nuttin's self-reflection paradigm. The FIQT has three major advantages over existing measures of self-reflection. First, it removes the need for participants to imagine themselves in an evaluative context, because it is an evaluative context. Second, because all participants perform the same, their perceptions of their performance reflect trait-like individual differences in the positive or negative valence of their self-reflection, rather than differences in other attributes. Third, because self-reflection is elicited by an external event, responses should be less influenced by variations in life history. Thus, the FIQT test could provide a useful alternative to existing measures of self-reflection. Further, validation in participants with MDD could provide a better understanding of the neural correlates of pathological self-reflection.

## Aims

To test the psychiatric validity of the FIQT, two experiments were conducted in participants with MDD and unaffected controls. The first aimed to pilot the computerised version of the FIQT to assess self-reflection in participants with MDD and controls, and explore construct validity through correlations with previously validated self-report measures of self-reflection. We hypothesised that participants with MDD would show higher levels of negative self-reflection on the FIQT compared with controls. It was also hypothesised that negative self-reflection on the FIQT would positively correlate with self-reported rumination, self-criticism and state worry, and negatively correlate with self-reassurance. The second experiment aimed to pilot the functional magnetic resonance imaging (fMRI) version of the FIQT, to examine neural activation during self-reflection in those with MDD compared with controls. We hypothesised increased activation in the medial prefrontal cortex (PFC), posterior cingulate cortex (PCC) and insula (reflecting self-reflection^13–16^), as well as the dorsolateral PFC and dorsal anterior cingulate cortex (ACC) (reflecting error processing^8,17,18^) in self-reflection versus control trials. We also hypothesised that this increased activation would be greater in participants with MDD compared with controls. An additional aim was to examine whether neural activity was related to scores on FIQT subscales and existing self-report measures of self-reflection. We hypothesised that elevated medial PFC, PCC, dorsal ACC, insula and dorsolateral PFC activation would be positively correlated with FIQT subscale scores and self-reported self-criticism, rumination and state worry, and negatively correlated with self-reassurance scores.

## Method

### Experiment 1

#### Participants

Participants aged 18–65 years were recruited via waiting lists for South London psychological therapy services and online advertisements. Participants had not yet started psychological therapy at the time of testing. Participants were required to meet the DSM-IV criteria for a current major depressive episode, determined via the Mini-International Neuropsychiatric Interview (MINI),^19^ and a score ≥14 on the 17-item Hamilton Rating Scale for Rating Scale (HRSD-17).^20^ A diagnosis of bipolar disorder or current psychosis (assessed with the MINI), or borderline personality disorder (determined via the Structured Clinical Interview for DSM-IV Axis II disorders^21^), were exclusionary.

Age- and gender-matched controls were assessed to exclude personal and familial (first-degree relative) psychiatric history. Exclusion criteria for all participants included neurological disorders, intellectual disabilities, visual problems that were not correctable, illicit substance use in the preceding 2 months, current (within 12 months of study entry) alcohol or other substance misuse (determined via the MINI), and unstable or severe medical conditions.

#### Ethics

This paper describes work submitted as part of the first author's PhD thesis^22^ and material has been included with permission. The authors assert that all procedures contributing to this work comply with the ethical standards of the relevant national and institutional committees on human experimentation and with the Helsinki Declaration of 1975, as revised in 2008. All procedures involving human patients were approved by London-Bromley Research Ethics Committee (reference: 13/LO/1897). All participants provided informed written consent and received financial compensation for taking part.

#### Materials

##### The FIQT

**Fig. 1:**
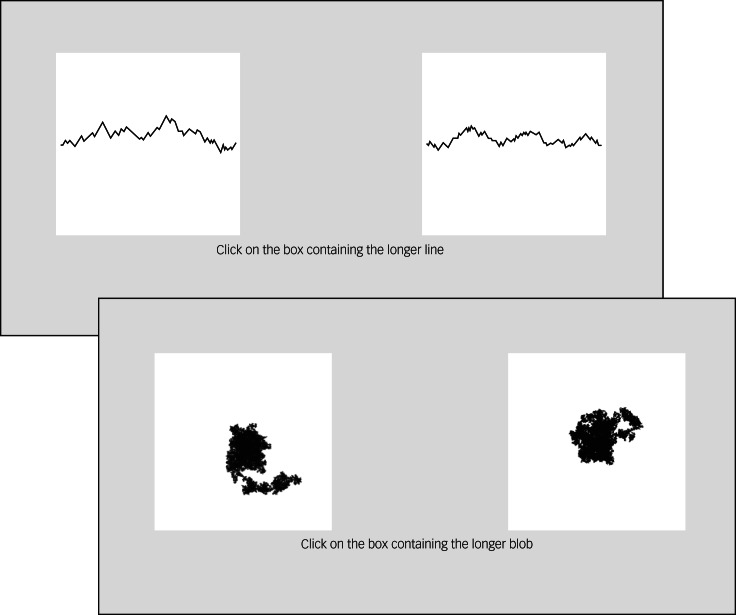
Example stimuli from the Fake IQ Test.

Participants read a brief statement before beginning the task, which informed them that the task was an examination of their visual perception, purposefully designed to be challenging and test their analytical ability. The construct supposedly being measured is fictitious but was described to sound plausible to the lay person, thus increasing individuals’ expectations that the task is linked to intellectual ability and performance, on which they would be judged. Participants were instructed to make quick and accurate judgements about predefined properties of pairs of geometric shapes displayed side by side on the computer screen. This visual perception performance on the task was not measured as all problems were impossible in that the two geometric shapes were equivalent in terms of the criteria the participant had to judge them on; for example, length, surface area or volume (see Fig. 1). Participants had 5.5 s to respond to each pair of images. After a set of ten images, participants were asked to rate their performance on three dimensions, using visual analogue scales: accuracy (‘How many of the last 10 trials do you think you got correct?’), comparison with others (‘Do you think your performance was better or worse than average?’) and satisfaction (‘Overall, do you feel satisfied with your performance?’). Scores on these questions were reversed (so that higher scores reflected more negative self-reflection), giving three subscale scores of ‘incorrect’, ‘comparison’ and ‘dissatisfaction’, respectively.

After completion of the FIQT, two further questions were asked: ‘Is how you performed on this task important to you?’ (rated from 0 ‘not at all’ to 10 ‘very important’) and ‘How did you feel during the task?’ These were asked to ensure potential differences in task performance were not a result of differences in motivation or guessing the ‘fake’ nature of the task.

##### Questionnaires

Three questionnaires were administered. The Rumination Response Scale (RRS)^23^ is a 22-item self-report measure of rumination, self-reflection and brooding. Higher scores indicate more ruminative symptoms. The Forms of Self Criticising/Attacking and Self-Reassuring Scale (FSCSR)^24^ is a 22-item self-report questionnaire exploring tendencies to be self-critical or self-reassuring to personal setbacks and failures. We used the two subscale scores of self-criticism and self-reassurance. The Penn State Worry Questionnaire (PSWQ)^25^ is a 16-item self-report questionnaire measuring trait worry. Higher scores indicate a higher tendency for worry.

#### Analysis

Differences in demographic and clinical variables and behavioural performance on the FIQT were assessed with independent samples *t*-tests. Associations between FIQT subscales, self-report measures of self-reflection (RRS, FSCSR and PSWQ) and depression severity (HRSD-17) were assessed with Pearson's correlations, using false discovery rate (FDR) correction.^26^

### Experiment 2

#### Participants

A subset of participants who took part in experiment 1 also took part in experiment 2, with additional exclusion criteria: psychotropic medication use in the 8 weeks before inclusion, pregnancy, left-handedness or other contraindications for scanning.

#### Materials

**Fig. 2:**
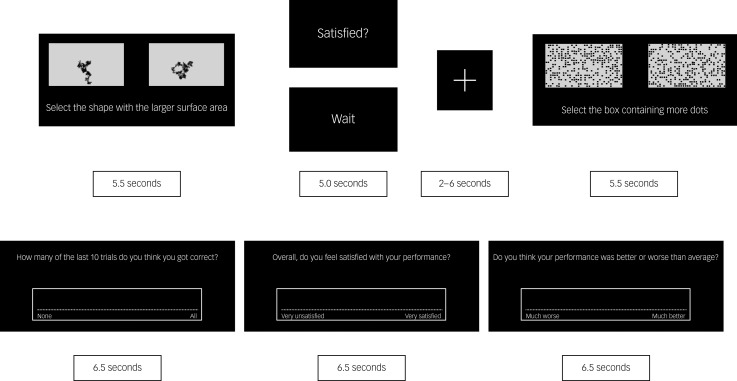
Stimulus timings for the functional magnetic resonance imaging version of the Fake IQ Test.

In the fMRI version of the FIQT, participants saw a screen after each pair of images, saying either ‘wait’ or ‘satisfied’ for 5 s (see Fig. 2). On the ‘satisfied’ trials, participants were instructed to reflect on their perceived performance on that trial (self-reflection trial). On the ‘wait’ trials, the participants were instructed to not think about their performance, but rather rest, relax and try to free their mind from the task (control trial). A white cross was then shown for a variable interstimulus interval of 2–6 s. ‘Wait’ and ‘satisfied’ trials were presented in a randomised order. Again, blocks of trials were followed by a performance rating. There were four blocks for each FIQT subscale performance rating.

Participants completed the RRS, FSCSR and PSWQ as in experiment 1.

#### Analysis

Differences in demographic and clinical variables and behavioural performance on the FIQT were assessed with independent samples *t*-tests. To examine whether performance changed over time, multiple linear regression analyses were run to test whether block and group significantly predicted FIQT subscale scores.

#### fMRI acquisition and pre-processing

Functional images were acquired on a 3-Tesla GE MR750 scanner with a 12-channel radiofrequency head coil. The fMRI sequence comprised T2*-weighted gradient echo planar image sessions of 338 whole-brain volume acquisitions: flip angle 75°, repetition time 2000 ms, echo time 30 ms, field of view 24 × 24 cm, slice thickness 3 mm, inter-slice gap 0.3 mm (total of 41 slices) and matrix size 64 × 64 voxels with an isotropic 3 mm × 3 mm in-plane resolution. Cardiac and respiratory signals were also recorded during the scan, and were processed with a custom script implementing the AFNI program's Retrospective Image Correction tool (RETROICOR) algorithm.^27,28^

Data pre-processing was conducted with custom Nipype scripts (http://nipy.org/nipype/), using tools from Statistical Parametric Mapping, version 12 for Windows (SPM12, UCL, London, UK; see http://www.fil.ion.ucl.ac.uk/spm/) and custom code. Functional images were realigned, slice-time corrected and co-registered to a high-resolution T1 image. T1 images were segmented and normalised, and functional images were normalised into Montreal Neurological Institute (MNI) space with deformation fields. Data were then smoothed with a 6 mm full-width half-maximum Gaussian kernel. To limit the effect of motion artefacts, participants with substantial translation of more than one voxel (3 mm) were removed from further analysis. Additionally, volumes with high levels of motion (based on realignment parameters and signal intensity changes from volume to volume) were identified with ArtifactDetect (see https://nipype.readthedocs.io/en/latest/api/generated/nipype.algorithms.rapidart.html), and removed from analysis.

#### First- and second-level analysis

First-level models were formed on each participant's data to generate mean images for each participant that included regressors for each trial type (‘wait’ (control condition), ‘satisfied’ (self-reflection condition) and baseline fixation), along with six motion parameters that were generated during realignment, and cardiac and respiratory regressors. Motion-scrubbing regressors were included to exclude volumes with high motion.

The contrast of interest for our main effects analyses compared ‘wait’ and ‘satisfied’ trials (controlling for fixation, which was modelled as an implicit baseline condition). Group differences between these conditions were tested with one-sample *t*-tests in SPM12, with head motion (total distance travelled), age and gender as covariates.

Regions of interest (ROIs) were derived from Neurosynth maps (see neurosynth.org) for the term ‘self-referential’, thresholded at *z* > 10 to identify regions most likely to be associated with self-reflective thought. This map was combined with the Automated Anatomical Labelling (AAL version 1 for Windows; see https://www.gin.cnrs.fr/en/tools/aal/) atlas^29^ to create the ROIs and additionally eliminate non-default mode network (DMN) regions from these maps. Regions within default mode systems, especially the medial PFC and PCC, were selected because these regions are considered imperative to the generation of self-referential thoughts.^30^ The ROI analyses were conducted by using separate masks for each ROI that were combined and treated as a single ‘small volume’ for the purposes of multiple comparison correction. For these bidirectional ROIs, the significance level for the F-contrasts were set to *P* < 0.05 FDR-corrected. As this was the first use of the FIQT, exploratory whole-brain analyses were also performed, with a cluster-defining threshold of *P* < 0.001 and a cluster-wise threshold of *P* < 0.05 FDR-corrected for multiple comparisons.

Regressions were conducted to explore relationships between neural activation (‘satisfied’ compared with ‘wait’ conditions) and self-report measures of self-reflection. These included the FIQT's subscale scores, self-criticism (FSCSR, self-criticism subscale), self-reassurance (FSCSR, self-reassurance subscale), rumination (RRS) and worry (PSWQ) questionnaire scores. These regressions were conducted at both a whole-brain level and on a mask of the main effect of the task (i.e. running correlations only in the areas that were significantly activated in the task) to explore correlations between behavioural scores and neural activity. This limited our analyses of relationships with questionnaires to relevant regions and ensured that the analyses were unbiased, because of the orthogonal nature of the primary activation analyses and those targeting relationships with questionnaires.

## Results

### Experiment 1

Thirty participants with MDD and 20 controls took part in experiment 1. Demographic and clinical information as well as FIQT and self-report questionnaire scores are displayed in Table 1. As expected, participants with MDD estimated that they made more mistakes (*P* = 0.011, Cohen's *d =* 0.81), perceived their performance as worse compared with others (*P* = 0.005, Cohen's *d =* 0.90) and were more dissatisfied with their performance (*P* = 0.048, Cohen's *d =* 0.61). The groups did not differ in post-task ratings of importance of performing well on the task.

**Table 1:** Experiment 1: mean (s.d.) sample characteristics, Fake IQ Test and self-report self-reflection scores

	Major depressive disorder (*n* = 30)	Controls (*n* = 20)	Test statistics
Age, years	35.2 (13.1)	32.0 (10.1)	*t*(48) = 0.9, *P* = 0.360
Male/female, *n*	9/21	7/13	*χ*^2^(1) = 0.14, *P* = 0.710
HRSD-17	19.4 (4.0)	0.95 (1.4)	*t*(48) = 19.8, *P* < 0.001
FIQT, Incorrect	54.1 (21.1)	40.6 (10.3)	*t*(48) = 2.7, *P* = 0.011
FIQT, Comparison	56.9 (18.5)	43.9 (8.5)	*t*(48) = 2.9, *P* = 0.005
FIQT, Dissatisfaction	54.4 (22.3)	43.2 (12.8)	*t*(48) = 2.0, *P* = 0.048
FIQT, Importance	6.6 (2.4)	5.4 (2.8)	*t*(48) = 1.6, *P* = 0.112
FSCSR, Self-Reassurance	12.5 (6.0)	25.6 (3.4)	*t*(48) = –8.8, *P* < 0.001
FSCSR, Self-Criticism	36.6 (4.0)	13.1 (7.9)	*t*(48) = 13.9, *P* < 0.001
RRS	66.8 (9.9)	31.4 (7.5)	*t*(48) = 13.6, *P* < 0.001
PSWQ	65.9 (7.5)	34.8 (11.8)	*t*(48) = 11.3, *P* < 0.001

MDD, major depressive disorder; HRSD-17, 17-item Hamilton Rating Scale for Depression; FIQT, Fake IQ Test; FSCSRS, Forms of Self-Criticising and Self-Reassuring Scale; RRS, Ruminative Response Scale; PSWQ, Penn State Worry Questionnaire.

Significant positive correlations were observed between all subscales of the FIQT in both groups (Table 2). However, no significant correlations were found between the FIQT subscales and self-reported self-reflection or depression severity measures in either group. Finally, no participants reported fully guessing the ‘fake’ nature of the task.

**Table 2:** Experiment 1: correlations between all self-reflection assessments and depression severity, by group (major depressive disorder in lower half of matrix, controls in upper half)

Variable	1	2	3	4	5	6	7	8
1 FIQT, Incorrect	−	0.87***	0.76***	−0.01	−0.33	−0.16	0.28	0.01
2 FIQT, Comparison	0.94***	−	0.82***	0.11	−0.35	−0.18	0.25	0.01
3 FIQT, Dissatisfaction	0.81***	0.88***	−	−0.28	−0.16	0.02	0.01	−0.14
4 FSCSRS, Self-Reassurance	0.03	0.01	−0.13	−	−0.51*	−0.07	−0.27	−0.37
5 FSCSRS, Self-Criticism	0.19	0.24	0.26	−0.65***	−	0.37	0.68***	0.73***
6 HRSD-17	−0.04	0.08	0.12	−0.35	0.29	−	0.47*	0.07
7 RRS	0.11	0.10	0.10	−0.26	0.40*	0.25	−	0.64***
8 PSWQ	−0.08	−0.11	0.00	0.09	0.29	0.06	0.11	−

FIQT, Fake IQ Test; FSCSRS, Forms of Self-Criticising and Self-Reassuring Scale; HRSD-17, 17-item Hamilton Rating Scale for Depression; RRS, Ruminative Response Scale; PSWQ, Penn State Worry Questionnaire.

*P < 0.05, ***P < 0.001.

### Experiment 2

Eighteen participants with MDD and 20 controls took part in experiment 2. However, three participants (two with MDD and one control) were excluded from analyses because of excessive head motion. Demographic and clinical information for included participants are shown in Table 3. Participants with MDD scored significantly higher than controls on all subscales of the FIQT apart from dissatisfaction, where there was a trend for participants with MDD to score higher than controls.

**Table 3:** Experiment 2: mean (s.d.) sample characteristics and behavioural performance on the Fake IQ Test

	Major depressive disorder (*n* = 16)	Controls (*n* = 19)	Test statistics
Age, years	32.5 (11.5)	32.2 (10.2)	*t*(33) = 0.1 *P* = 0.944
Male/female, *n*	6/10	7/12	*χ*^2^(1) = 0.00, *P* = 0.968
HRSD-17	19.1 (3.8)	0.7 (1.1)	*t*(33) = 19.7, *P* < 0.001
FSCSR, Self-Criticism	33.7 (9.5)	12.7 (7.9)	*t*(33) = 7.3, *P* < 0.001
FSCSR, Self-Reassurance	13.5 (5.6)	25.6 (3.5)	*t*(33) = −7.9, *P* < 0.001
RRS	67.6 (7.7)	30.6 (6.7)	*t*(33) = 13.8, *P* < 0.001
PSWQ	66.9 (7.5)	33.8 (11.3)	*t*(33) = 9.7, *P* < 0.001
FIQT, Incorrect	53.7 (14.6)^a^	40.2 (10.5)	*t*(32) = 3.1, *P* = 0.004
FIQT, Comparison	54.7 (14.7)^a^	43.7 (8.6)	*t*(32) = 2.7, *P* = 0.010
FIQT, Dissatisfaction	53.4 (18.7)^a^	43.0 (13.1)	*t*(32) = 1.9, *P* = 0.066

HRSD-17, 17-item Hamilton Rating Scale for Depression; FSCSRS, Forms of Self-Criticising and Self-Reassuring Scale; RRS, Ruminative Response Scale; PSWQ, Penn State Worry Questionnaire; FIQT, Fake IQ Test.

a. *n* = 15 because of data collection errors for one participant.

Regression analyses indicated that group significantly predicted FIQT incorrect (*β* = 12.55, *P* < 0.001), comparison (*β* = 11.01, *P* < 0.001) and dissatisfaction scores (*β* = 10.38, *P* = 0.001); however, trial block did not (incorrect: *β* = 0.52, *P* = 0.672; comparison: *β* = 1.84, *P* = 0.098; satisfied: *β* = 1.39, *P* = 0.323).

**Fig. 3:**
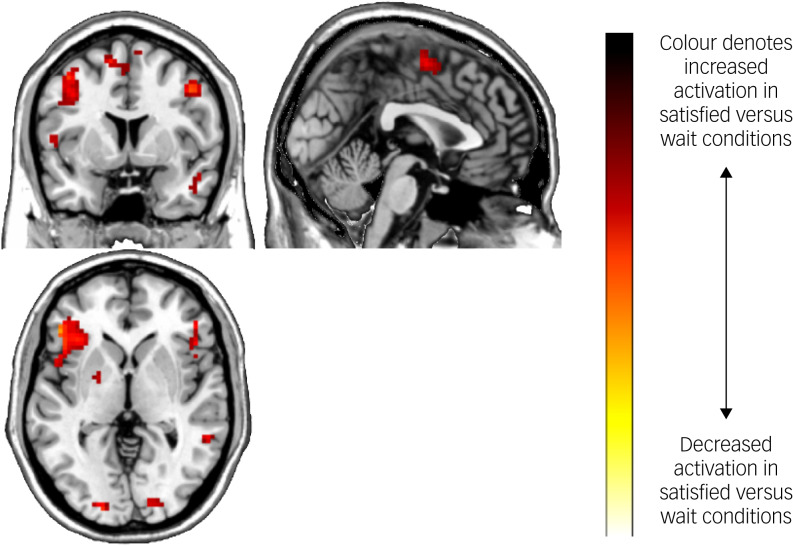
Brain activation during the Fake IQ Test (satisfied > control conditions).

Task main effects for the whole-brain analysis are displayed in Table 4 and Fig. 3. There was increased activation in the self-reflection versus control condition bilaterally in the inferior cortex extending to the dorsal ACC and insula, as well as the left dorsolateral PFC, and motor area activation. There were no significant areas of decreased activation with self-reflection. There were no significant differences between groups in the whole-brain analysis.

**Table 4:** Experiment 2: brain activation during the Fake IQ Test (main effect of task)

Brain regions	Peak MNI coordinate	*P-*values of peak cluster	Voxels	F	Direction of activation
Left inferior occipital/left mid occipital cortex	−15, −91, −7	0.006	89	63.43	Satisfied > wait condition
Left inferior frontal gyrus/dorsolateral prefrontal cortex/insula	−48, 32, −1	<0.001	486	38.62	Satisfied > wait condition
Left inferior parietal cortex	−51, −49, 47	0.010	76	29.22	Satisfied > wait condition
Right frontal inferior gyrus, slightly into right insula	51, 26, 5	0.006	90	27.85	Satisfied > wait condition
Left supplementary motor area/left frontal superior cortex	−12, 5, 62	0.006	92	25.91	Satisfied > wait condition

*N* = 35 (16 participants with depression, 19 controls), whole-brain analysis. All results presented met criteria for significance; a cluster-defining significance of *P* < 0.001 and a cluster-wise threshold of *P* < 0.05 FDR-corrected. MNI, Montreal Neurological Institute.

No significant effects of task or group were found in our ROI analysis. The regression analyses did not show any significant relationships between brain activity (at the whole-brain level or the main effect of task) and self-report measures of self-reflection or behavioural subscales of the FIQT.

## Discussion

In accordance with our hypothesis, participants with MDD showed higher levels of negative forms of self-reflection on all subscales of the FIQT in experiment 1. Although group differences in scores on the dissatisfaction subscale became non-significant in experiment 2, this is likely because of the smaller sample size and consequent reduction in statistical power. Interestingly, we did not find any significant associations between FIQT subscales and self-reported self-reflection or depression severity in either group. In experiment 2, increased activation was found in the insula, dorsal ACC and dorsolateral PFC in self-reflection versus control trials of the FIQT; however, no differences in activation were found in those with MDD compared with controls. Further, no significant effects of task or group were found in our ROI analysis, and brain activity did not correlate with FIQT subscales or self-report measures of self-reflection.

Our findings showing increased negative self-comparison with others, increased dissatisfaction and lower perceived success on the FIQT are consistent with previous research showing high levels of self-criticism, low self-esteem and heightened perception of failure in those with MDD.^12,31^ Participants with MDD and controls gave similar post-task ratings of task importance, suggesting group differences were not a result of controls caring less about performance, but are reflective of differences in self-perception of performance. These findings suggest that the FIQT may be sensitive to behavioural differences in self-reflection in MDD, providing a novel, objective measure of negative self-appraisal.

Given the lack of relationships between FIQT subscales and self-report measures of self-reflection, the FIQT may be tapping into aspects of self-reflection inaccessible to current questionnaires. The FIQT relies less on insight and does not require recall of memories with potentially high emotional content, unlike the questionnaire methods used in this study. Further, the non-verbal nature of the task may partly explain the lack of association with self-report measures. However, these characteristics may make the FIQT more widely accessible to groups with special requirements (for example, young children or individuals with autism). On the other hand, the lack of construct validity here may indicate the FIQT measures a different construct entirely. Future research should carefully consider the psychological correlates of the FIQT. For example, previous work has suggested that self-criticism may mediate the relationship between maladaptive perfectionism and psychological distress.^32^ Future work could evaluate whether the FIQT more closely reflects perfectionistic aspects of self-reflection.

Although those with MDD had higher scores on the FIQT compared with controls, we did not find any association between depressive symptoms and FIQT subscale scores. Further, no significant associations were found between FIQT subscale scores and neural activation, preventing us from interpreting whether regions of increased activation during self-reflection trials versus control trials related to negative forms of self-reflection. Given that negative self-reflection is considered a core element of depression, our findings may indicate that the FIQT measures a neurocognitive process or type of self-reflection not linked to severity of mood. MDD is typically characterised by negative ruminative thoughts about the self (e.g. ‘I am worthless/inadequate/to blame’), whereas the type of self-reflection elicited in the FIQT is relatively emotionally neutral. Thus, the lack of association with depressive symptoms may be because of these qualitative differences in self-reflective content. Future studies could explore qualitative differences in thoughts surrounding the FIQT, which may reveal important differences across disorders.

Increased activation in the insula, dorsal ACC and dorsolateral PFC was found in self-reflection versus control trials of the FIQT. These results are consistent with previous research implicating these areas in self-reflection and error processing.^8,15,17,18^ Additionally, significantly elevated activation in the self-reflection condition was found in the posterior inferior parietal lobe. This region is part of the DMN and is associated with attending to visual, spatial stimuli.^33^ The observed activation in inferior, occipital and parietal cortices, along with the dorsal ACC and insula, could reflect the increased emotional salience of the self-reflection trials. These regions form part of the ‘salience network’, which is associated with the processing of salient, internally generated emotional thoughts.^34^ The insula mediates the detection of motivationally salient and emotional stimuli,^35^ and the salience network has been found to have reciprocal connections and causally influence activity in the DMN.^34^ We also found elevated motor activation during self-reflection, likely reflecting participants imagining the shapes and engaging in motor imagery.^36^

Currently, psychiatric assessment and diagnosis is based on self-report and observation of patient behaviours, without parallel measurement of underlying biological mechanisms. However, clinical presentation varies widely within psychiatric diagnoses, suggesting that there are multiple underlying causes and illness processes. Recent neuroimaging studies have suggested that neurophysiological-based subtypes of disorders may be better able to predict treatment response and symptoms than traditional classification systems, although further replication of these findings are required^37,38^ As self-reflection is considered a spectrum, with both high and low levels found in a range of psychopathologies, then a better understanding of the neural correlates of specific aspects of self-reflection may inform us about the development and maintenance of disordered thinking and allow for more personalised treatments.

### Limitations

The control condition of the FIQT, where participants were instructed to relax, could still elicit self-reflection. This would be supported by findings that the DMN is active in resting-state studies, where participants often report being engaged in self-reflective thought.^39^ This may explain why, contrary to our expectations, there were no differences in activation between the control and self-reflective conditions in the medial PFC and PCC, areas that have been previously identified as active during self-reflection.^13,14,16^ This could additionally explain why our ROI analyses did not reveal any significant task effects, because of our focus on DMN regions in selection of these regions. Despite evidence of differences between resting-state and task-based self-reflective neural activation, many self-reflection studies do use similar control conditions to the FIQT and have found DMN activation.^40^ Relatedly, our lack of group differences in neural activation may have been because of differences in participants’ abilities to shift their attention between control and self-reflective trials. For example, participants with MDD may have been less able to relax and free their mind of negative thoughts during the control condition, confounding group differences. The relatively short trial duration may have exacerbated this issue.

Our lack of significant group differences in experiment 2 may indicate that the FIQT is not sensitive to the neural correlates of psychopathology. However, further exploration of the task is warranted, given that other studies have found differences in neural activation in depression during negative self-reflection,^7,41^ and self-reflective tasks have been shown to be sensitive to both pharmacological^42^ and psychological therapeutic response.^43^ Compared with tasks used previously, the FIQT has the added benefit of not relying on insight or imagined states of self-reflection. However, the task could be adapted to test different control conditions; for example, by including a distractor (e.g. hand-tapping or neutral thought cues) to minimise self-reflective cognitions.

Scores on each of the FIQT subscales were highly correlated. This may indicate that the different questions were measuring the same construct. Finally, the relatively small sample sizes in both experiments are also a limitation, particularly for the development of a new measurement tool. This may have also prevented detection of significant differences.

In conclusion, our study presents behavioural and fMRI pilot testing of a novel, objective measure of self-reflection, the FIQT. Our results suggest the FIQT is sensitive to affective psychopathology with elevated negative self-comparison with others, higher self-dissatisfaction and lower perceived success in participants with MDD relative to controls. Importantly, the ‘fake’ nature of the task did not appear to have been perceived by participants in post-task questioning and evaluation of responses over time. Because of a lack of significant correlations between FIQT subscales and self-reported self-criticism, rumination and worry, we did not find evidence of construct validity. It is therefore possible that the FIQT does not measure self-reflection or may assess aspects of self-reflection not captured by current measures. Neural main effects showing elevated activation in the insula, dorsal ACC, dorsolateral PFC and posterior inferior parietal lobe provide further support for the FIQT as an assessment of self-reflection. We therefore propose the FIQT as an alternative to traditional self-report measures of self-reflection, with potential application to multiple patient groups. Further work should explore the task's relationship to alternative measures of self-reflection likely to be involved in perception of task performance, such as self-blame or perfectionism.

## Data Availability

The data that support the findings of this study are available from the corresponding author, J.K.-G., upon reasonable request.
